# Fissure of the Anus

**Published:** 1871-12

**Authors:** 


Liniment for Fissure of the Anus—M. Van Halsbeck states that he has succeeded in curing anal fissures with the following application which had resisted the division of the sphincter: Dissolve one part of tannic acid in 16 parts of glycerine. A tent wet with this preparation is to be introduced into the rectum night and morning. The bowels are to be kept open.—*Medical News.*

